# Precision oncology: Charting a path forward to broader deployment of genomic profiling

**DOI:** 10.1371/journal.pmed.1002242

**Published:** 2017-02-21

**Authors:** Alison M. Schram, Michael F. Berger, David M. Hyman

**Affiliations:** 1 Department of Medicine, Memorial Sloan Kettering Cancer Center, New York, New York, United States of America; 2 Department of Pathology, Memorial Sloan Kettering Cancer Center, New York, New York, United States of America; 3 Weill Cornell Medical College, New York, New York, United States of America

## Abstract

In this Perspective, David Hyman and coauthors discuss clinical research on the application of genomic information in oncology.

Over the past 15 years, we have seen a dramatic shift in the delivery of clinical care in oncology. The profound impact of trastuzumab in treatment of HER2-amplified breast cancer and of imatinib in chronic myeloid leukemia harboring the BCR-ABL translocation sparked intensive efforts to reproduce these early successes by targeting therapy to a tumor’s individual molecular profile. This increased focus on precision medicine has grown in parallel with exponential improvements in the sequencing technologies used to decipher the cancer genome. High-throughput next-generation sequencing (NGS) has made it possible to rapidly and accurately detect all classes of genomic alterations in tumors, including sequence mutations, copy number alterations, and structural rearrangements in hundreds or even thousands of genes simultaneously [1–3]. As the sophistication of testing has improved, sharp declines in cost have facilitated access in the clinic.

The appeal of large gene panels has been driven by the operational efficiencies afforded by running one standardized assay instead of multiple single analyte or customized gene panels, improved sensitivity compared to traditional methodologies, and the desire to detect rare and potentially actionable genomic variants across cancer types [3]. Broad NGS can also provide additional clinically important information such as identification of microsatellite instability (MSI) and hypermutation, acquired resistance mutations, and, in assays utilizing paired germline sequencing, the presence of cancer predisposition alleles [4–6]. This convergence of factors is expected ultimately to make NGS more cost-effective than performing multiple parallel tests.

Tumor sequencing is already used to guide routine treatment decisions in many cancers including non-small cell lung cancer, melanoma, colorectal cancer, gastrointestinal stromal tumors, ovarian cancer, and several leukemias. Genomically guided therapies have produced durable responses in these diseases [7]. What remains unanswered is how broadly applicable this paradigm will be to the overall population of cancer patients. To date, many large NGS efforts have resulted in disappointingly low rates of enrollment into clinical trials designed to test responses to genomically guided treatments [8,9]. Careful analysis of these data suggests that this low rate reflects a clinical research system that is ill-suited to provision of rational therapeutic options for patients profiled by large-panel NGS assays rather than the absence of potentially actionable alterations detected by this technology. Major barriers to genomically matching patients include the availability of relevant trials, physician and patient knowledge of these studies, and geographical access to studies. Moreover, the diversity of tumor types that can harbor potentially targetable alterations, as well as the rarity of many of these alterations within any given tumor type, have made traditional disease-specific studies difficult to conduct.

Studies that define eligibility based on the presence of a specific genetic alteration irrespective of histology, so called “basket trials,” have emerged as one particularly efficient way to treat patients with genomically matched therapy and generate high-quality evidence to guide future treatment. The design of these basket studies also acknowledges that response to targeted therapy can be conditioned by tumor type, further reinforcing the importance of generating such data. Recognizing the value of this approach, ambitious efforts such as the National Cancer Institute’s Molecular Analysis for Therapy Choice (NCI-MATCH) study have been launched that combine genomic screening with a large selection of therapeutic studies assigned based on the profiling results. However, despite plans to sequence tumor DNA from 5,000 patients, several of the treatment arms in NCI-MATCH enrolling less common variants are not expected to accrue sufficient numbers of patients [10]. This further emphasizes that successfully conducting studies in the rarest variants will ultimately require even broader adoption of large-panel NGS uncoupled from individual therapeutic studies.

Experience using large NGS panels for mutation profiling has already led to the detection of rare genomic alterations across tumor types and improved treatments for patients harboring these alterations. For example, fusions in the genes encoding the three isoforms of tropomyosin receptor kinase proteins (NTRK1/2/3) have been found in nearly a dozen distinct tumor types to date, albeit at low frequency, and have led to near-universal, dramatic, and durable responses to selective tyrosine kinase inhibitors in clinical trials [11]. These early clinical trial data led the FDA to grant breakthrough therapy designation to one compound for the treatment of “metastatic solid tumors with NTRK-fusion proteins,” raising the possibility of the first entirely genomics-driven, tumor type–agnostic drug approval [12]. Similarly, ALK and ROS1 fusions arising in diseases other than lung cancer have been identified in multiple tumor types and demonstrate very promising sensitivity to targeted therapy [13]. Without the availability of broad NGS panel testing, individuals with these alterations and other patients would miss the opportunity to be treated with effective therapy.

A critical question for justifying the long-term adoption of this technology is determining the scope of patients who harbor actionable alterations and therefore may benefit from genomically matched therapy. Previously reported clinical sequencing efforts have found that 30%–80% of advanced solid tumors harbor potentially actionable alterations [2,8]. However, many of these alterations have not been clinically validated and remain under investigation. Additionally, we believe that binary classification of actionable alterations oversimplifies interpretation of data. Nevertheless, as research progresses, novel therapies are developed, and new clinical trials become available, the number of actionable alterations will continue to increase.

Furthermore, the utility of NGS goes beyond identifying aberrations that are predictive of response to a targeted therapy and may also predict response to immunotherapy by identifying MSI or other hypermutated phenotypes, provide important diagnostic or prognostic information, and identify treatment-informing biomarkers that predict a lack of drug response. Leveraging the tremendous amount of genomic data generated by NGS to improve clinical care will require a multidisciplinary approach involving physicians, scientists, computational biologists, statisticians, drug companies, and patients. Data sharing across institutions and laboratories engaged in this testing is essential and will allow us to identify patterns and explore hypotheses that will otherwise be impossible. The American Association of Cancer Research (AACR) recently introduced Project GENIE, an international data-sharing venture that will aggregate clinical-grade cancer genomic data with clinical outcomes initially across eight major cancer centers. The Global Alliance for Genomics and Health is also engaged in an ambitious effort to pool genomic and clinical data across various stakeholders [14].

Currently, large panel testing is considered investigational in all but a small number of cancers and is therefore generally not reimbursed by third party payers. This lack of reimbursement has been a major barrier to the adoption of NGS sequencing technology and consequently our ability to evaluate its utility. While payers understandably expect proof that this testing improves patient outcomes overall, developing this critical evidence base will require screening of very large numbers of patients. Therefore, we find ourselves in a catch-22. Large academic institutions have relied heavily on grants and philanthropic contributions to support internal sequencing efforts, yet this is not sustainable over the long term. This cost must be shared among all stakeholders and the outcomes carefully measured. Further, our ability to evaluate the clinical utility of molecular profiling depends on broadening access to clinical trials for patients treated outside of academic medical centers. To address this need, several novel study designs are emerging to bring the study to the patient. The Signature study, for example, utilized a “just-in-time” model of study activation to bring matched targeted therapy to patients utilizing any available genomic testing [15]. The American Society of Clincal Oncology (ASCO) recently launched its first clinical trial, Targeted Agent and Profiling Utilization Registry (TAPUR) (www.tapur.org), which provides free matched therapy of marketed drugs to providers and collects a minimal dataset on outcome.

We believe a period of broad access to testing is necessary to accumulate a knowledge base sufficient to evaluate the clinical utility of biomarkers representing not only common genetic alterations in common diseases but also rarer alterations and diseases. We have a responsibility to patients to pursue the promise afforded by this potentially revolutionary technology. Importantly, containing costs will require close partnership with industries to improve the likelihood that insights gained from sequencing will be pursued therapeutically. Collaborations, multisite clinical trials, and data sharing are all necessary to maximize knowledge acquired from sequencing efforts. While we pursue this ambitious agenda, it is important that we continually revisit assumptions regarding the clinical benefit of precision medicine to determine if expectations are being met. Only then can we truly judge the value of precision oncology.

## Abbreviations

MSI: microsatellite instability
NGS: next-generation sequencing
